# EXIT25 – Executive interview applied to a cognitively healthy elderly population with heterogeneous educational background

**DOI:** 10.1590/S1980-57642009DN20400013

**Published:** 2008

**Authors:** Maria Niures P.S. Matioli, Paulo Caramelli, Bárbara D. Marques, Fernanda D. da Rocha, Maria Cristina C. de Castro, Samia R. Yamashita, Alberto de M. Soares

**Affiliations:** 1 From the Department of Geriatrics, Department of Internal Medicine, Faculty of Medicine of the Federal University of Minas Gerais, Belo Horizonte (MG), Brazil.; 2 Lusíada University School of Medicine, Santos (SP), and the Behavioral and Cognitive Neurology Unit, Department of Internal Medicine, Faculty of Medicine of the Federal University of Minas Gerais, Belo Horizonte (MG), Brazil.

**Keywords:** EXIT25, executive functions, aging, education, cognitive tests

## Abstract

Education interferes with the performance in most cognitive tests, including executive function assessment.

**Objective:**

To investigate the effects of education on the performance of healthy elderly on the Brazilian version of the Executive Interview (EXIT25).

**Methods:**

The EXIT25 was administered to a sample of 83 healthy elderly. The subjects were also submitted to the Mini-Mental State Examination (MMSE), a delayed recall test, clock drawing and category fluency (animals/min) tests in order to rule out cognitive impairment. The Geriatric Depression Scale (GDS) was employed to exclude clinically-relevant depressive symptoms. The total sample was divided into three groups according to educational level: G1 (1–4 years), G2 (5–8 years) and G3 (>8 years).

**Results:**

The mean values for age, educational level, MMSE and EXIT25 scores of all subjects were 72.2, 7.5, 27.6 and 6.9, respectively. The scores on the EXIT25 for each group were: G1=8.3, G2=5.9 and G3=5.8. There was a statistical difference between the performance of G1 and the other two groups on the EXIT25.

**Conclusions:**

The Brazilian version of the EXIT25 proved straightforward to administer. The performance of this sample of healthy elderly on the test was significantly influenced by educational level.

## Abstract (pt)

O nível educacional interfere no desempenho da maioria dos testes cognitivos incluindo os de função executiva.

**Objetivo:**

Investigar os efeitos da educação no desempenho de idosos saudáveis na versão brasileira do teste de funções executivas (EXIT25).

**Métodos:**

O EXIT25 foi administrado a uma amostra de 83 idosos saudáveis. Os indivíduos foram também submetidos ao Mini-Exame de Estado Mental (MEEM), a um teste de evocação, ao desenho do relógio e ao teste de fluência de categorias (animais/min) com a função de excluir prejuízos cognitivos. A Escala de Depressão Geriátrica foi aplicada para excluir sintomas de depressão clinicamente relevante. O total da amostra foi dividido em três grupos de acordo com o nível educacional: G1 (1–4 anos), G2 (5–8 anos) e G3 (>8 anos).

**Resultados:**

As médias de idade, nível educacional, MEEM e EXIT25 de todos os indivíduos foram respectivamente de 72,2; 7,5; 27,6 e 6,9. Os valores do EXIT25 para cada grupo foram: G1=8,3; G2=5,9 e G3=5,8. Houve diferença estatística entre o desempenho de G1 e os outros dois grupos no EXIT25.

**Conclusões:**

A versão brasileira do EXIT25 provou ser de fácil administração. O desempenho da amostra de idosos examinada foi significativamente influenciada pelo nível educacional.

The executive functions are defined by the Diagnostic and Statistical Manual of Mental Disorders, fourth edition (DSM-IV) as an individual’s ability to plan, initiate, sequence, monitor, and inhibit complex behavior.^1^ These functions are crucial to maintain independent living, involving tasks such as dressing, cooking, housework or self-care, where, devoid of these functions, patients become dependent and may also present behavioral problems.^2,3^

Royall et al.^4^ described two main caveats for defining executive function: the first associates executive function with “higher” cognitive functions (e.g., insight, abstraction, will and judgment) mostly dependent on functioning of the frontal lobe, while the second emphasizes behavioral regulation of non-executive processes by frontal control systems.^5^

The prefrontal cortex and related subcortical structures consist of striatal-cortical-frontal control circuits that are associated with specific executive functions.^6^ These circuits run from the orbitofrontal, dorsolateral, and medial prefrontal cortex to the striatum, proceeding to the globus pallidus and thalamus, returning to the prefrontal cortex.^5^ Many neurotransmitters are involved in these circuits, especially dopamine.^7^ Impairment of executive functions can be due to either gray or white matter damage in these circuits.^5^ Many neurological and psychiatric disorders are associated with frontal cortico-subcortical impairments such as dementia associated with Parkinson’s disease, subcortical vascular dementia, depression and schizophrenia.^8-11^

Royall et al.^2^ developed a clinically-based bedside screening instrument to determine deficits in these domains, namely the *Executive Interview (EXIT25)*. This test is easily and quickly applied (requiring approximately 15 minutes) and can also be administered by properly trained non-medical personnel.

The EXIT25 consists of 25 items:

1) number-letter task;
2) word fluency (letter “A”);
3) design fluency;
4) anomalous sentence repetition;
5) thematic perception;
6) memory/distraction task;
7) interference task;
8) automatic behavior I;
9) automatic behavior II;
10) grasp reflex;
11) social habit ;
12) motor impersistence;
13) snout reflex;
14) finger-nose-finger task;
15) go/no-go task;
16) echopraxia I;
17) Luria hand sequence I;
18) Luria hand sequence II;
19) grip task;
20) echopraxia II;
21) complex command task;
22) serial order reversal task;
23) counting task;
24) utilization behavior;
25) imitation behavior.

Each item of the EXIT25 is scored as: 0=intact performance; 1=specific partial error or equivocal response; 2= specific incorrect response or failure to perform the task. Global scores range from 0 to 50, with high scores indicating executive impairment. Royall et al.^2,12^ found that a score of 10/50 reflects the 5^th^ percentile for young adults and scores ≥15/50 suggest significant executive dysfunction.

The EXIT25 has high interrater reliability and correlates well with other measures of executive function, such as the Wisconsin Card Sorting Task – Categories, Trail Making Test Part B, Lezak’s Tinker Toy Test and the Test of Sustained Attention.^2,12^

The clinical applications of the EXIT25 are manifold. The test can be used for assessing executive functions in elderly people with normal cognition or with cognitive impairment, to identify specific subtypes of mild cognitive impairment and the risk of dementia conversion, as well as in the context of the authors’ initial idea, i.e., to establish functional status, self-care deficits and to predict problematic behaviors or psychiatric disturbances of demented subjects.^2,12-15^

In the present study, we administered the Brazilian version of the EXIT25 to a sample of healthy elderly people in order to determine its applicability and the extent to which education influences EXIT scores.

## Methods

Initially, we evaluated a random sample of 111 literate (one year or more of schooling) elderly subjects, aged 60 years and older attended at the Geriatric Outpatient Clinic of Guilherme Álvaro Hospital in Santos, Brazil, together with individuals drawn from the community. The study was approved by the Ethics Committee of the hospital and all subjects signed a written informed consent.

None of the participants had history of cognitive or psychiatric symptoms and all were submitted to clinical and neurological examinations to exclude disorders that could have influenced cognitive performance. Moreover, those taking medications with central nervous system action were also excluded. The DSM-IV^1^ was used as a reference to rule out dementia and psychiatric disorders. The Geriatric Depression Scale (GDS)^16^ (with 30 questions) was employed as a screening instrument for ruling out clinically relevant depressive symptoms, indicated by scores ≥10.

All subjects were submitted to the following brief cognitive tests to ensure normal cognitive status: the Mini-Mental State Examination (MMSE),^17,18^ delayed recall test of 10 simple figures,^19,20^ the clock drawing test ^21^ and category fluency (animals/min.).^22^ Cut-off scores for the MMSE were defined according to educational level: <22 points for individuals with 1 to 3 years of schooling; <24 for those with 4 to 7 years; <26 for subjects with 8 years or more.^18^ The cut-off scores for the remaining tests were: <6 for delayed recall of the 10 simple figures;^19,20^ < 7 for clock drawing; and <10 animals/min. in the category fluency test for individuals with ≤4 years of schooling, and <13 for those with ≥5 years.^23^ Subjects showing impairment on at least one cognitive test were excluded.

The total sample was divided into three groups according to educational level: G1 (1–4 years), G2 (5–8 years) and G3 (>8 years), according to the division adopted when the elderly subjects went to school.

The EXIT25 was translated and adapted to the Portuguese (Brazilian) language following a strict methodology, which included two translations from English into Portuguese by two independent translators (one geriatrician and one neurologist), followed by back translation. The test was administered by a previously trained team comprising a neurologist, two geriatricians and four undergraduate students of Medicine from the Lusíada University School of Medicine (UNILUS). The students received the same training for the application of the EXIT25, by the neurologist and the geriatricians who also supervised the clinical and neurological examination.

Statistical analysis employed the Kruskal-Wallis test to compare the sociodemographic variables among the three groups. The Mann-Whitney test was applied to determine differences between pairs of groups. All statistical tests were interpreted at the 5% significance level. Univariable correlations of Spearman’s coefficient between EXIT25 and each cognitive measure were applied to the total sample. The multivariable linear regression model was used to determine the contribution of the independent variables gender, age, education and GDS performance to EXIT25 scores.

## Results

From the initial sample of 111 subjects, 28 (25.2%) were excluded: 27 showed impairment on at least one cognitive test and one had GDS=12.

The final sample was composed of 83 individuals (74.8%), comprising 35 men with mean age and schooling of 71.4 (SD=6.9) and 8.3 (SD=5.4) years, respectively; and 48 women with mean age and schooling of 72.8 (SD=6.7) and 6.9 (SD=4.3) years, respectively. There were no statistical differences regarding age (p=0.401) and schooling (p=0.258) between the two genders.

Mean age, schooling and MMSE scores for the total sample were 72.2 (SD=6.8), 7.5 (SD=4.8) and 27.6 (SD=1.6), respectively. The MMSE average score was 27.8 (SD=1.5) for the men and 27.5 (SD=1.6) for the women, with no significant differences between these (p=0.289).

Sociodemographic characteristics of the sample along with results of the cognitive tests applied are shown in Table 1.

**Table 1:** Main sociodemographic characteristics and results of the cognitive tests: total sample, Groups 1, 2 and 3.

Variable	Total sample N=83 F=48 M=35 Mean (SD)	Group 1 Schooling 1-4 years N=38 F=23 M=15 Mean (SD)	Group 2 Schooling 5-8 years N=16 F=11 M=5 Mean (SD)	Group 3 Schooling >8 years N=29 F=14 M=15 Mean (SD)	G1×G2×G3
Age	72.2 (6.8)	73.4 (7.4)	72.6 (6.1)	70.1 (6.2)	p=0.162
MMSE	27.6 (1.6)	26.9 (1.7)	28.1 (1.4)	28.2 (1.1)	p=0.005
GDS	4.2 (2.6)	5.1 (2.4)	3.7 (3.3)	3.6 (2.4)	p=0.040
Verbal Fluency Animals/min	15.3 (3.3)	14.1 (2.9)	15.9 (3.5)	16.8 (3.0)	p<0.001
Clock drawing	9.3 (0.8)	9.1 (0.8)	9.6 (0.5)	9.4 (0.8)	p=0.072
Delayed recall test of 10 figures	8.3 (1.3)	8.2 (1.2)	8.2 (1.4)	8.5 (1.4)	p=0.529

N, sample size; F, female; M, male; G1, Group 1; G2, Group 2; G3, Group 3; SD, standard deviation; MMSE, Mini-Mental State Examination; GDS, Geriatric Depression Scale.

The EXIT25 test was easily administered and took 15 minutes on average to apply. Mean score for the global sample was 6.9 (SD=3.0). Significant differences between the performance of men and women on the EXIT25 was observed, with men presenting worse performance than women (7.9±2.9vs. 6.2±2.9, respectively, p=0.006).

The performance of Group 1 on the EXIT25 (mean=8.3, SD=3.2) was significantly worse than Groups 2 (mean=5.9, SD=2.6) (p=0.035) and 3 (mean=5.8, SD=2.9) (p=0.001), although there was no significant difference between Groups 2 and 3 (p=0.537).

The sample was subdivided into two subgroups in order to evaluate the possible influence of age on performance: 60 to 70 years (n=35) and older than 70 years (n=48). These two groups had similar educational level and MMSE scores. No difference was observed between these two subgroups in relation to performance on the EXIT25 (p=0.133). Table 2 shows the results of EXIT25 scores.

**Table 2:** Scores on the EXIT25 by Groups 1, 2 and 3.

	Group 1 Schooling 1-4 years N=38 F=23 M = 15 Mean (SD)	Group 2 Schooling 5-8 years N=16 F=11 M=5 Mean (SD)	Group 3 Schooling >8 years N=29 F=14 M=15 Mean (SD)
EXIT25	8.3 (±3.2)	5.9 (±2.6)	5.8 (±2.9)
G1×G2×G3	G1×G2	G1×G3	G2×G3
p=0.002	p=0.035	p=0.001	p=0.537

N, sample size; F, female; M, male; G1, Group 1; G2, Group 2; G3, Group 3; SD, standard deviation.

The EXIT25 scores correlated with all cognitive measures: MMSE (r= –0.38; p=0.0004), category fluency (r= –0.37; p=0.0005), clock drawing (r= –0.27; p=0.0138) and delayed recall of the 10 simple figures (r= –0.26; p=0.0185). Multivariable linear regression considering gender, education, age and GDS performance as independent variables showed that gender (r= –.33; p<0.001) and education (r= –.41; p<0.001) were significantly correlated with EXIT25 scores.

## Discussion

The EXIT25 proved to be swift and straightforward to administer, and applicable by non-medical trained personnel such as the assisting undergraduate medical students in this study. The test was not administered to illiterate elderly since the test was devised for educated individuals where many of the test items assume literacy.

Men performed worse than women on the Brazilian version of the EXIT25. Several studies in which executive functions were assessed using other tests have also described different performances between males and females.^24-26^ However, we shall consider that the present result may represent an anomaly due to the relatively small sample size.

The heterogeneous educational level of the Brazilian population, especially among the elderly, led us to divide the total sample into three groups. The different EXIT scores obtained across all three groups, especially for those with less than 5 years of schooling compared to G2 and G3 groups, suggest that educational level constitutes an important variable influencing performance on this test. Previous studies have reported correlation among performances on executive function tests and educational level in the elderly.^23,24,26-30^ Hashimoto at al.^31^ stated that the effects of education on executive function in normal elderly subjects was unclear.

The original EXIT25 research was conducted with 40 elderly subjects, randomly selected from residents of an extended care community in San Antonio, Texas (USA), where individuals were divided into two groups: a non-institutionalized group (N=20) having normal cognitive function, and an institutionalized group (N=20). The non-institutionalized group had higher education (12.8 (2.6) and also higher scores on the EXIT25 (14.2 (7.5) compared to our data. Royall et al.^12-14,30^ have been studying executive control function of elderly retirees (N=193) without dementia for three years, recruited from a randomly ordered list of Air Force Villages from the Freedom House Study (FHS). At baseline, the FHS subjects had a higher level of education (15.1 years) and worse performance on the EXIT25 (14.6) in comparison to our results. In contrast to the current authors, investigators in both of the above studies had not divided their samples into different levels of schooling. We believe the possible reason behind the score diferential observed between our data and both original and FHS Royall et al. studies, is the nature of the samples. The present sample was composed of elderly subjects requiring no care or supervision and selected from the general community, whereas the cited studies involved residents of an extended care community.^2,12^

It is important to mention several limitations of our study. The main constraint was the small sample size and the difficulty in finding subjects with 5 to 8 years of schooling (Group 2), since most literate elderly people in Brazil have either completed four years of schooling and then interrupted their formal education, or continued their studies to 8 years or more (especially among men), in order to complete a technical course or to go to University.

In conclusion, we found that educational level significantly influenced the performance of cognitively healthy elderly subjects on the EXIT25. Further studies including larger samples of elderly individuals with and without cognitive impairment, will allow different education-adjusted cut-off scores to be determined for the EXIT25 in our milieu.
